# Influence of oral tobacco versus smoking on multiple sclerosis disease activity and progression

**DOI:** 10.1136/jnnp-2022-330848

**Published:** 2023-03-31

**Authors:** Jing Wu, Tomas Olsson, Jan Hillert, Lars Alfredsson, Anna Karin Hedström

**Affiliations:** 1 Aging Research Center, Department of Neurobiology, Care Sciences and Society, Karolinska Institutet and Stockholm University, Stockholm, Stockholm, Sweden; 2 Department of Clinical Neuroscience, Karolinska Institutet, Stockholm, Sweden; 3 Institute of Environmental Medicine, Karolinska Institutet, Stockholm, Sweden; 4 Centre for Occupational and Environmental Medicine, Region Stockholm, Stockholm, Sweden

**Keywords:** MULTIPLE SCLEROSIS, QUALITY OF LIFE

## Abstract

We aimed to study the influence of smoking habits, exposure to passive smoking and snuff use on disease progression, cognitive performance and quality of life in patients with multiple sclerosis (MS).

**Method:**

Patients from two population-based case–control studies were categorised based on tobacco exposure at diagnosis and were followed up to 15 years post diagnosis through the Swedish MS registry (n=9089) regarding changes in Expanded Disability Status Scale (EDSS), Multiple Sclerosis Impact Scale 29 and Symbol Digit Modalities Test. We used linear mixed models to analyse long-term changes, and Cox regression models with 95% CI using 24-week confirmed disability worsening, reaching EDSS 3 and EDSS 4, respectively, physical and psychological worsening and cognitive disability worsening as end points. The influence of smoking cessation post diagnosis was also investigated.

**Results:**

Compared with non-smokers, current smokers had a faster EDSS progression (β_current smoking×time_=0.03, 95% CI 0.02 to 0.04). A faster EDSS progression was also associated with passive smoking (β_current passive smoking×time_=0.04, 95% CI 0.03 to 0.06). Smoke exposure negatively impacted all secondary outcomes. Those who continued smoking had worse outcomes than those who stopped smoking post diagnosis. Snuff users had a more favourable EDSS progression, compared with never users.

**Conclusions:**

Our findings indicate that both smoking and passive smoking have a negative influence on MS and that smoking cessation post diagnosis may be an important secondary preventive measure. Snuff use was associated with slower disease progression, suggesting that nicotine replacement therapy could be an attractive way to increase the chance of quitting smoking among patients with MS.

**WHAT IS ALREADY KNOWN ON THIS TOPIC**

- Smoking negatively impacts the severity and progression of multiple sclerosis (MS), whereas the possible influence of exposure to passive smoking versus the influence of nicotine in the form of moist snuff on MS progression has not previously been investigated.

**WHAT THIS STUDY ADDS**

- Smoking negatively affects MS in a dose-dependent manner.
- Among never smokers, exposure to passive smoking was associated with faster disease progression, a higher risk of physical and psychological worsening and a higher risk of cognitive disability worsening.
- The use of moist snuff was significantly associated with slower disease progression.

**HOW THIS STUDY MIGHT AFFECT RESEARCH, PRACTICE OR POLICY**

- Nicotine replacement therapy could be an attractive way to increase the chance of successful smoking cessation among patients with MS who smoke.

## Background

Several studies have shown that smoking negatively impacts the severity and progression of multiple sclerosis (MS), an immune-mediated disorder of the central nervous system. Smokers have been found to have higher Expanded Disability Status Scale (EDSS) and Multiple Sclerosis Severity Score (MSSS) scores than non-smokers,^1 2^ and increased risk of reaching disability milestones, increased MRI activity and greater brain atrophy.^2–4^ Associations have also been reported between smoking and fatigue, depressive symptoms and lower health-related quality of life in patients with MS.^5^ Continued smoking after MS diagnosis has consistently been associated with faster disease progression, whereas other studies have observed deceleration of the disease progression following smoking cessation.^6–8^

Since smoking is a modifiable risk factor in MS, cessation of smoking is important. However, several MS-specific barriers to smoking cessation have been identified in qualitative studies, and patients have expressed insecurity about whether nicotine replacement therapy would be safe in MS or in combination with MS disease-modifying treatment.^9–11^ Moist snuff is a traditional Scandinavian smokeless tobacco product to place behind the upper lip. Snuff use delivers high amounts of nicotine without airway irritation and has not been associated with the risk of developing MS,^12 13^ but the possible impact of nicotine on MS disease progression has not previously been investigated. This is also the case for passive smoking, which is associated with increased MS risk, but has not been evaluated against progression.

We followed up 9089 patients with MS from two case–control studies through the Swedish MS registry to investigate the impact of smoking (including smoking cessation), exposure to passive smoking and snuff use, using clinical and self-reported outcome measures.

## Methods

Our study comprises patients from the two population-based case–control studies Epidemiologic Investigation of Multiple Sclerosis (EIMS) and Genes and Environment in Multiple Sclerosis (GEMS). Incident cases were recruited to EIMS from hospital-based neurology units between April 2005 and December 2019 (n=3567), whereas GEMS identified prevalent cases, distinct from those in EIMS, from the Swedish MS registry between November 2009 and November 2011 (n=6148). The response rate among cases was 93% in EIMS and 82% in GEMS. All cases fulfilled the McDonald criteria.^14 15^ More details on study design and methods are given elsewhere.^16^ Of the 9715 patients, 9089 (94%) were followed-up with EDSS scores in the Swedish MS registry.

As a complement, to explore the influence of lifestyle changes after diagnosis on disease activity and progression, patients included in EIMS between April 2005 and December 2019 were asked to fill out a digitalised follow-up questionnaire regarding lifestyle habits, covering the time period between diagnosis and 2021. Completed questionnaires were provided by 1823 patients (66%), of which 1733 (95%) were followed up with EDSS in the Swedish MS registry.

### Definition of exposures

Information on environmental exposures and lifestyle habits was collected at study inclusion using a standardised questionnaire. Information on smoking habits at diagnosis was obtained by asking about current and previous smoking, including duration of smoking (time periods) and intensity of smoking (average number of cigarettes smoked per day, for each time period). In the EIMS follow-up questionnaire, participants were asked whether they had smoked after the diagnosis of MS, and if they had, they were asked to state during which time period/periods as well as the frequency of smoking (average number of cigarettes per day). Smoking was categorised into never smoking, past smoking or current smoking at the time of MS diagnosis. Smokers completing the EIMS follow-up questionnaire were further categorised into those who had continued versus stopped smoking 5 years post diagnosis.

Passive smoking history was captured by asking about exposure to environmental tobacco smoke at home or at work. Those who reported daily exposure to environmental tobacco smoke at home or work were defined as exposed to passive smoking. The patients were categorised into never, past or current exposure to passive smoking at diagnosis. Snuff use was defined as regular use of moist snuff and patients were divided into never, past or current snuff users at the time of diagnosis.

### Outcome measures

In accordance with recommendations by the Swedish MS association, detailed information is continuously and routinely registered in the national Swedish MS registry regarding medical treatment, disease activity, physical functioning, as well as mental health and quality of life.

In order to study changes in severity/disability over time, baseline was defined as the date of the first recorded EDSS at the time of diagnosis or later. Confirmed disability worsening (CDW) was defined as an increase in the EDSS Score with at least 1 point from baseline sustained between two follow-up visits separated in time by no less than 6 months (1.5 points if EDSS at baseline was 0, 0.5 points if the baseline EDSS≥5.5). Time to milestones EDSS 3 and 4 were studied as secondary outcomes and were limited to subgroups of patients with a baseline EDSS of less than 3. Other secondary outcomes were change in health-related quality of life as measured by the Multiple Sclerosis Impact Scale 29 physical score (MSIS-29-PHYS).^17^ An increased score of 7.5 points or more in the physical and mental components, respectively, of MSIS-29 was defined as a worsening from the patient’s perspective. This cut-point is recommended for identifying patients with clinically significant change in the physical impact of MS.^18^ The Symbol Digit Modalities Test (SDMT) was used as a measure of cognition performance outcome.^19^ An 8-point or greater worsening of the SDMT, which has been suggested for identifying meaningful cognitive decline,^20^ was defined as cognitive disability worsening.

### Statistical analysis

Categorical variables were summarised using frequency and percentage. Continuous variables were summarised using mean and SD or median and IQR as appropriate.

The associations between smoking and passive smoking, respectively, and level at baseline (intercept) and rate of change over 15 years (slope) in MS progression scores (EDSS/MSIS-PHYS/SDMT) were estimated using linear mixed effect models. Never smokers and those never exposed to passive smoking were treated as the reference group. To consider the non-linear change over time, we introduced a quadratic term of follow-up time (years from the first assessment) in the models and the non-linearity was tested by Wald test. Random intercept and slope were estimated as the random part in the mixed effect model. The participants were grouped on the patient ID. The unstructured covariance matrices were used for all models. The estimated trajectories of MS progression scores by smoking status were constructed by plotting marginal prediction of the sample mean from the mixed models during the follow-up and reported at key time points (0, 3, 5, 10 and 15 years) and the model specification is presented in the online supplemental file.

10.1136/jnnp-2022-330848.supp1 Supplementary data

Time to 24-week CDW and the secondary milestones EDSS 3 and 4 end points as well as physical worsening from the patient’s perspective and cognitive disability worsening were analysed using multivariable Cox proportional hazard regression. The follow-up time was calculated as the time from the baseline until the onset of the events of interest, drop-out, death or end of follow-up (6 April 2022), whichever occurred first. The proportional hazard assumption was tested through the Schoenfeld residuals. No violations of proportionality were observed. A trend test for a dose–response relationship regarding the cumulative dose of smoking and unfavourable outcomes was performed by using a continuous variable in a Cox regression model. The influence of smoking on disease outcomes was also analysed by sex. We further studied the influence of passive smoking on disease progression restricted to never smokers.

All analyses controlled for age at diagnosis, sex, disease phenotype, disease duration (time between clinical onset and MS diagnosis), baseline EDSS and disease-modifying therapy (ever or never). The following potential confounding variables were not kept in the final model since they had a minor influence on the results: alcohol consumption at diagnosis (no consumption, low, moderate or high consumption according to cut-offs used by Statistics Sweden^21^) and body mass index at diagnosis (underweight, normal weight, overweight or obese, as defined by the WHO^22^).

Several supplementary analyses were performed. Since the majority of patients receive disease-modifying treatment, which has a profound influence on disease outcomes, we performed subanalyses in which we excluded subjects who were untreated. We also performed a subanalysis only comprising participants who had started treatment with disease-modifying drugs within 6 months after diagnosis. Since some clinics may have followed-up patients with MS digitally during the COVID-19 pandemic, we also performed the analysis using January 2020 as the end of follow-up, before the outbreak of the pandemic. We carried out analyses considering smoking habits at the time of disease onset. Finally, we performed the analysis for EIMS and GEMS participants separately.

All analyses were conducted in Stata V.17.0 (StataCorp, Texas, USA) and Statistical Analysis System V.9.4.

## Results

Our study comprised 9089 patients with MS. The mean age at baseline was 37.6 years (SD11.1) and 6541 (72%) were females. Baseline characteristics of cases, overall and by smoking, passive smoking and snuff use status, respectively, are presented in online supplemental tables 1–3.

### Smoking and MS progression

**Figure 1:**
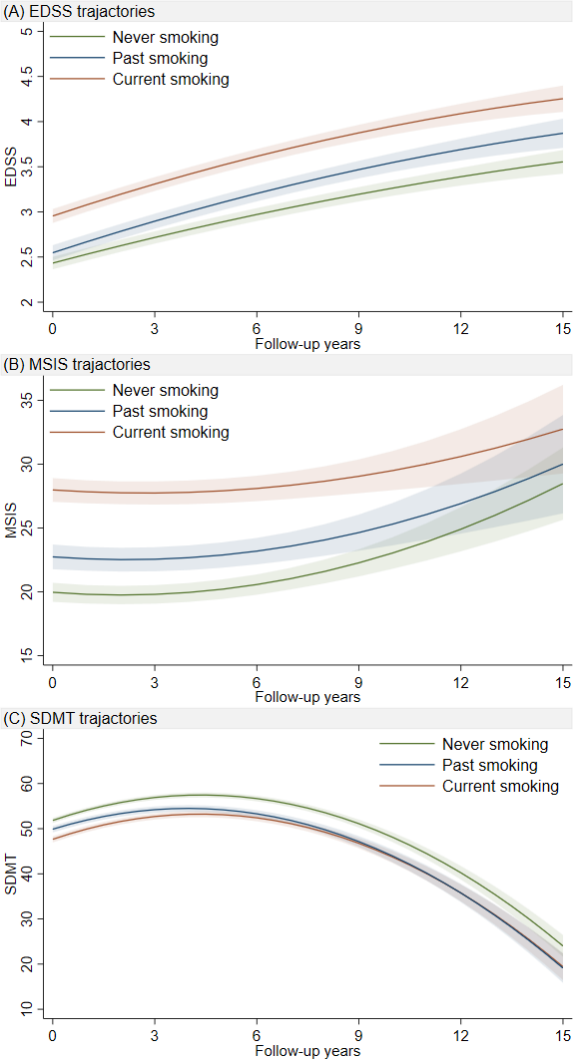
Predicted trajectories of EDSS, MSIS-PHYS and SMDT scores over a 15-year period post diagnosis, by smoking status at diagnosis. Based on data in online supplemental table 5. The lines represent β coefficients from the linear mixed effects model adjusted for sex, duration between onset-diagnosis, disease-modifying treatment, age at diagnosis, disease phenotype and baseline EDSS. Models considered a non-linear trajectory with quadratic time (with corresponding random effects). EDSS, Expanded Disability Status Scale; MSIS-PHYS, Multiple Sclerosis Impact Scale physical score; SMDT, Symbol Digit Modalities Test.

At baseline, current smokers had significantly higher EDSS scores (0.48, 95% CI 0.38 to 0.58) compared with non-smokers (figure 1, online supplemental tables 4–5). In comparison to never smokers, we observed a faster increase in EDSS both for current smokers (β_current smoking×time_=0.03, 95% CI 0.02 to 0.04) and past smokers (β_past smoking×time_=0.02, 95% CI 0.01 to 0.03).

At baseline, higher MSIS-PHYS scores were observed both among current smokers (6.61, 95% CI 5.43 to 7.80) and past smokers (1.21, 95% CI −0.02 to 2.44), while the rates of MSIS-PHYS change were stable over time (p>0.05) (figure 1, online supplemental tables 4–5).

Current smokers had a lower SDMT score at baseline (−3.64, 95% CI −4.50 to –2.78), and past smoking was associated with a faster decrease in SDMT scores (β_past smoking×time_=−0.27, 95% CI −0.43 to –0.10) during follow-up compared with never smoking (figure 1, online supplemental tables 4–5).

Similarly, compared with never smoking at baseline, current smoking was associated with an increased risk of developing CDW (adjusted HR 1.13, 95% CI 1.06 to 1.21), and an increased risk of reaching EDSS 3 (adjusted HR 1.21, 95% CI 1.09 to 1.34) and EDSS 4 (adjusted HR 1.31, 95% CI 1.14 to 1.51), whereas past smoking at baseline was not significantly associated with these outcomes (table 1). Current smoking was also associated with a higher risk of physical and psychological worsening, as well as cognitive disability worsening than never smokers (table 1). We observed trends showing increased risk of unfavourable outcomes with cumulative dose of smoking regarding all outcomes (table 1). Our findings remained similar or were slightly higher among men after stratification by sex (online supplemental table 6).

**Table 1:** HR with 95% CI of having unfavourable outcomes post diagnosis, by smoking habits at diagnosis

Smoking status	N	Years (SD)	Outcome (%)	HR (95% CI)*	aHR (95% CI)†
First clinical disease worsening					
Never smoking	3675	6.5 (4.9)	2031 (55)	1.0 (reference)	1.0 (reference)
Past smoking	2517	6.4 (4.9)	1435 (57)	1.06 (0.99 to 1.13)	1.01 (0.94 to 1.08)
Current smoking	2897	6.5 (5.1)	1760 (61)	1.13 (1.06 to 1.20)	1.13 (1.06 to 1.21)
Cumulative dose of smoking (unit: pack-year)	9089	6.4 (5.0)	5226 (58)	1.06 (1.04 to 1.07)	1.03 (1.01 to 1.05)
EDSS 3					
Never smoking	2281	7.9 (5.3)	867 (38)	1.0 (reference)	1.0 (reference)
Past smoking	1491	7.7 (5.3)	610 (41)	1.11 (1.00 to 1.23)	1.00 (0.90 to 1.12)
Current smoking	1469	8.0 (5.6)	678 (46)	1.23 (1.11 to 1.36)	1.21 (1.09 to 1.34)
Cumulative dose of smoking (unit: pack-year)	5241	7.9 (5.4)	2155 (41)	1.11 (1.07 to 1.14)	1.05 (1.02 to 1.08)
EDSS 4					
Never smoking	2281	9.7 (5.3)	466 (20)	1.0 (reference)	1.0 (reference)
Past smoking	1491	9.5 (5.4)	317 (21)	1.06 (0.92 to 1.23)	0.96 (0.82 to 1.10)
Current smoking	1469	10.1 (5.7)	397 (27)	1.28 (1.11 to 1.45)	1.31 (1.14 to 1.51)
Cumulative dose of smoking (unit: pack-year)	5241	9.7 (5.5)	1180 (23)	1.12 (1.07 to 1.16)	1.04 (1.00 to 1.08)
Physical worsening (increased MSIS-29 physical score by 7.5 or more)					
Never smoking	2114	5.2 (4.5)	891 (42)	1.0 (reference)	1.0 (reference)
Past smoking	1283	4.8 (4.2)	556 (43)	1.10 (0.99 to 1.22)	1.06 (0.95 to 1.19)
Current smoking	1324	5.0 (4.9)	627 (47)	1.19 (1.07 to 1.31)	1.23 (1.11 to 1.37)
Cumulative dose of smoking (unit: pack-year)	4721	5.0 (4.6)	2074 (44)	1.08 (1.05 to 1.11)	1.07 (1.03 to 1.10)
Psychological worsening (increased MSIS-29 psychological score by 7.5 or more)					
Never smoking	2109	4.8 (4.6)	1034 (49)	1.0 (reference)	1.0 (reference)
Past smoking	1274	4.4 (4.4)	663 (52)	1.14 (1.03 to 1.25)	1.18 (1.07 to 1.31)
Current smoking	1319	4.6 (4.9)	716 (54)	1.18 (1.08 to 1.30)	1.27 (1.15 to 1.40)
Cumulative dose of smoking (unit: pack-year)	4702	4.6 (4.9)	2413 (51)	1.04 (1.01 to 1.07)	1.08 (1.05 to 1.12)
Cognitive disability worsening (decreased SDMT Score by 8 or more)					
Never smoking	2217	5.5 (3.5)	504 (23)	1.0 (reference)	1.0 (reference)
Past smoking	1330	5.4 (3.5)	327 (25)	1.13 (0.99 to 1.30)	1.08 (0.93 to 1.25)
Current smoking	1390	5.6 (3.7)	315 (23)	1.00 (0.87 to 1.16)	1.17 (1.02 to 1.36)
Cumulative dose of smoking (unit: pack-year)	4937	5.5 (3.5)	1146 (23)	1.05 (1.01 to 1.09)	1.04 (1.00 to 1.09)

*Crude.

†Adjusted for age at diagnosis, sex, disease phenotype, disease duration, baseline EDSS, disease-modifying therapy, passive smoking and snuff use.

EDSS, Expanded Disability Status Scale; MSIS-29, Multiple Sclerosis Impact Scale 29; SDMT, Symbol Digit Modalities Test.

### Passive smoking and MS progression

Overall, 61% (n=5576) reported having been daily exposed to passive smoking before diagnosis. The mean duration of daily exposure was 15.9 years (SD 7.7).

**Figure 2:**
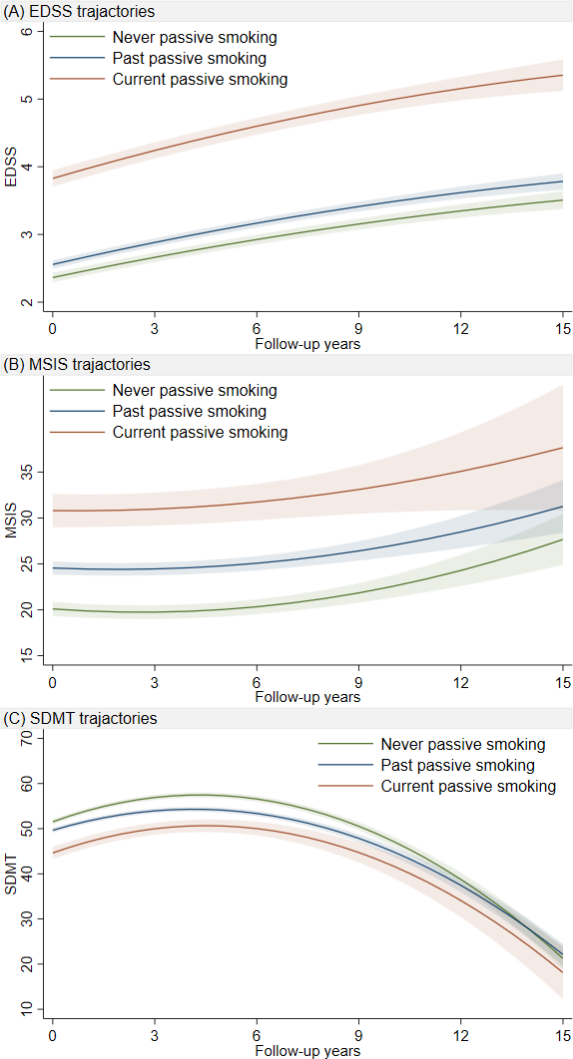
Predicted trajectories of EDSS, MSIS-PHYS and SDMT scores over a 15-year period post diagnosis, by passive smoking status at diagnosis. Based on data in online supplemental table 5. The lines represent β coefficients from the linear mixed effects model adjusted for sex, duration between onset-diagnosis, disease-modifying treatment, age at diagnosis, disease phenotype and baseline MSIS-29 physical score. Models considered a non-linear trajectory with quadratic time (with corresponding random effects). EDSS, Expanded Disability Status Scale; MSIS-PHYS, Multiple Sclerosis Impact Scale physical score; SMDT, Symbol Digit Modalities Test.

At baseline, a higher EDSS Score was significantly associated with current exposure to passive smoking (1.23, 95% CI 1.09 to 1.37) but not with previous exposure (0.05, 95% CI −0.04 to 0.15) compared with those who had never been exposed. A faster EDSS progression was observed both in those currently exposed to passive smoking (β_current passive smoking×time_=0.04, 95% CI 0.03 to 0.06) and in those previously exposed (β_past passive smoking×time_=0.01, 95% CI 0.001 to 0.02) during a 15-year follow-up (figure 2, online supplemental tables 4–5).

Higher MSIS-PHYS scores across the study period were also observed among both those with current and past exposure to passive smoking (7.67, 95% CI 5.70 to 9.64 and 2.00, 95% CI 0.93 to 3.06) compared with never exposure to passive smoking (figure 2, online supplemental table 4).

Those who were currently exposed to passive smoking had significantly lower SDMT scores at baseline (−5.83, 95% CI −7.27 to –4.40) and previous exposure was associated with faster decreasing SDMT (β_past passive smoking×time_=−0.49, 95% CI −0.63 to –0.36) during follow-up compared with those who had never been exposed (figure 2, online supplemental tables 4–5).

Compared with those who had never been exposed to passive smoking, current exposure was associated with an increased risk of CDW (adjusted HR 1.18, 95% CI 1.08 to 1.29), and an increased risk of reaching EDSS 3 (adjusted HR 1.49, 95% CI 1.29 to 1.74) and EDSS 4 (adjusted HR 1.79, 95% CI 1.48 to 2.15) (table 2). Current exposure to passive smoking was also associated with an increased risk of physical and psychological worsening, but not with cognitive disability worsening (table 2). Our findings remained significant when the analysis was restricted to never smokers (online supplemental table 7) and to those who received disease-modifying treatment (online supplemental table 8).

**Table 2:** HR with 95% CI of having unfavourable outcomes post diagnosis, by passive smoking status at diagnosis

Passive smoking	N	Years (SD)	Outcome (%)	HR (95% CI)*	aHR (95% CI)†
First clinical disease worsening					
Never exposed	3513	6.7 (5.0)	1972 (56)	1.0 (reference)	1.0 (reference)
Past exposure	4399	6.3 (4.9)	2517 (57)	1.08 (1.02 to 1.15)	1.02 (0.96 to 1.08)
Current exposure	1177	6.4 (5.1)	737 (63)	1.21 (1.11 to 1.32)	1.18 (1.08 to 1.29)
EDSS 3					
Never exposed	2223	8.3 (5.4)	853 (38)	1.0 (reference)	1.0 (reference)
Past exposure	2599	7.6 (5.4)	1076 (41)	1.17 (1.07 to 1.28)	1.03 (0.93 to 1.12)
Current exposure	419	7.4 (5.4)	226 (54)	1.59 (1.38 to 1.85)	1.49 (1.29 to 1.74)
EDSS 4					
Never exposed	2223	9.7 (5.5)	471 (21)	1.0 (reference)	1.0 (reference)
Past exposure	2599	9.5 (5.5)	556 (21)	1.13 (1.03 to 1.25)	0.94 (0.83 to 1.06)
Current exposure	419	9.4 (5.6)	153 (37)	1.86 (1.62 to 2.13)	1.79 (1.48 to 2.15)
Physical worsening (increased MSIS-29 physical score by 7.5 or more)					
Never exposed	2043	5.4 (4.5)	880 (43)	1.0 (reference)	1.0 (reference)
Past exposure	2334	4.8 (4.7)	1020 (44)	1.12 (1.03 to 1.23)	1.06 (0.96 to 1.16)
Current exposure	344	4.4 (4.3)	174 (51)	1.41 (1.20 to 1.66)	1.44 (1.22 to 1.70)
Psychological worsening (increased MSIS-29 psychological score by 7.5 or more)					
Never exposed	2038	4.9 (4.5)	1047 (51)	1.0 (reference)	1.0 (reference)
Past exposure	2323	4.5 (4.7)	1176 (51)	1.08 (1.00 to 1.18)	1.14 (1.04 to 1.24)
Current exposure	341	4.1 (4.3)	190 (56)	1.28 (1.10 to 1.50)	1.39 (1.19 to 1.63)
Cognitive disability worsening (decreased SDMT Score by 8 or more)					
Never exposed	2147	5.7 (3.5)	501 (23)	1.0 (reference)	1.0 (reference)
Past exposure	2431	5.3 (3.5)	567 (23)	1.12 (0.99 to 1.26)	1.03 (0.91 to 1.17)
Current exposure	359	5.7 (3.7)	78 (22)	1.00 (0.80 to 1.26)	1.17 (0.92 to 1.49)

*Crude.

†Adjusted for age at diagnosis, sex, disease phenotype, disease duration, baseline EDSS, disease-modifying therapy, smoking and snuff use.

EDSS, Expanded Disability Status Scale; MSIS-29, Multiple Sclerosis Impact Scale 29; SDMT, Symbol Digit Modalities Test.

### Snuff use and MS progression

After adjustment for potential confounding variables, including smoking and passive smoking, there was a borderline significant inverse association between snuff use and EDSS at baseline, compared with those who had never used snuff. Past snuff users scored significantly higher on SDMT compared with never users, whereas current users did not (online supplemental table 3).

Compared with those who had never used snuff, past snuff use was significantly associated with a lower risk of CDW (HR 0.84, 95% CI 0.73 to 0.97) and a lower risk of reaching EDSS 3 (HR 0.80, 95% CI 0.63 to 1.00) and EDSS 4 (HR 0.81, 95% CI 0.63 to 1.14). Current snuff use was significantly associated with a lower risk of reaching EDSS 4, but not with the risk of CDW or risk of reaching EDSS 3 (table 3). Snuff use was not significantly associated with the risk of health-related quality of life worsening or with the risk of cognitive disability worsening (table 3).

**Table 3:** HR with 95% CI of having unfavourable outcomes post diagnosis, by snuff use habits at diagnosis

Snuff use status	N	Years (SD)	Outcome (%)	HR (95% CI)*	aHR (95% CI)†	aHR (95% CI)‡	aHR (95% CI)§
First clinical disease worsening							
Never user	7521	6.4 (5.0)	4351 (58)	1.0 (reference)	1.0 (reference)	1.0 (reference)	1.0 (reference)
Past user	452	6.6 (5.0)	229 (51)	0.86 (0.75 to 0.98)	0.84 (0.74 to 0.96)	0.81 (0.71 to 0.93)	0.84 (0.73 to 0.97)
Current user	1097	6.5 (5.1)	634 (58)	0.97 (0.90 to 1.06)	0.89 (0.91 to 1.04)	0.96 (0.88 to 1.05)	0.87 (0.79 to 0.98)
EDSS 3							
Never user	4319	7.9 (5.3)	1807 (42)	1.0 (reference)	1.0 (reference)	1.0 (reference)	1.0 (reference)
Past user	268	7.6 (5.2)	84 (31)	0.77 (0.62 to 0.96)	0.79 (0.66 to 0.99)	0.76 (0.61 to 0.95)	0.80 (0.63 to 1.00)
Current user	647	7.9 (5.6)	260 (40)	0.94 (0.82 to 1.07)	0.95 (0.85 to 1.10)	0.91 (0.79 to 1.05)	0.85 (0.72 to 0.99)
EDSS 4							
Never user	4319	9.8 (5.5)	996 (23)	1.0 (reference)	1.0 (reference)	1.0 (reference)	1.0 (reference)
Past user	268	9.0 (5.1)	49 (18)	0.86 (0.64 to 1.10)	0.82 (0.71 to 1.04)	0.83 (0.64 to 0.12)	0.81 (0.63 to 1.14)
Current user	647	9.8 (5.8)	135 (21)	0.88 (0.74 to 1.05)	0.86 (0.62 to 1.09)	0.79 (0.66 to 0.97)	0.73 (0.58 to 0.92)
Physical worsening (increased MSIS-29 physical score by 7.5 or more)							
Never user	3789	5.0 (4.6)	1683 (44)	1.0 (reference)	1.0 (reference)	1.0 (reference)	1.0 (reference)
Past user	282	5.3 (5.2)	127 (45)	0.97 (0.81 to 1.16)	0.93 (0.77 to 1.11)	0.92 (0.76 to 1.10)	0.97 (0.79 to 1.19)
Current user	646	5.0 (4.0)	262 (41)	0.91 (0.80 to 1.04)	0.92 (0.80 to 1.06)	0.90 (0.79 to 1.05)	0.87 (0.74 to 1.02)
Psychological worsening (increased MSIS-29 psychological score by 7.5 or more)							
Never user	3775	4.7 (4.6)	1945 (52)	1.0 (reference)	1.0 (reference)	1.0 (reference)	1.0 (reference)
Past user	282	4.8 (4.9)	147 (52)	1.00 (0.84 to 1.18)	1.00 (0.84 to 1.19)	0.97 (0.83 to 1.12)	1.03 (0.88 to 1.28)
Current user	641	4.5 (3.9)	318 (50)	0.97 (0.86 to 1.09)	0.98 (0.87 to 1.12)	0.97 (0.86 to 1.11)	0.95 (0.82 to 1.10)
Cognitive disability worsening (decreased SDMT Score by 8 or more)							
Never user	3963	5.5 (3.6)	925 (23)	1.0 (reference)	1.0 (reference)	1.0 (reference)	1.0 (reference)
Past user	295	5.6 (3.4)	67 (23)	0.92 (0.72 to 1.18)	0.87 (0.69 to 1.12)	0.82 (0.63 to 1.06)	0.87 (0.65 to 1.15)
Current user	671	5.5 (3.6)	152 (23)	0.96 (0.81 to 1.14)	1.01 (0.88 to 1.22)	1.00 (0.83 to 1.20)	0.85 (0.69 to 1.05)

*Crude.

†Adjusted for age at diagnosis, sex, disease phenotype, disease duration, baseline EDSS, disease-modifying therapy, smoking and passive smoking.

‡Adjusted for age at diagnosis, sex, disease phenotype, disease duration, baseline EDSS and disease-modifying therapy.

§Restricted to participants who had initiated snuff use before disease onset.

EDSS, Expanded Disability Status Scale; MSIS-29, Multiple Sclerosis Impact Scale 29; SDMT, Symbol Digit Modalities Test.

The mean duration of snuff use was 9.4 years among current users and 17.8 years among past users. When we excluded snuff users who had initiated snuff use after disease onset (n=351; mean duration of snuff use 1.1 years), the HRs of EDSS-related outcomes became lower among current snuff users (table 3). Our findings remained significant when only patients who received disease-modifying treatment were included (online supplemental table 9).

### Continued smoking versus smoking cessation post diagnosis

Continued smoking after diagnosis was associated with a higher risk of CDW (HR 1.21, 95% CI 1.03 to 1.44), and a higher risk of reaching EDSS 3 (HR 1.45, 95% CI 1.16 to 1.81) and EDSS 4 (HR 1.44, 95% CI 1.10 to 1.89), compared with non-smokers at baseline (table 4). Continued smokers also had a higher risk of physical and psychological worsening and a higher risk of cognitive disability worsening than non-smokers. There was no significant difference between postdiagnosis quitters and non-smokers at baseline regarding any of these outcomes (table 4).

**Table 4:** HR with 95% CI of having unfavourable outcomes, by smoking habits post diagnosis (non-smokers, postdiagnosis quitters and postdiagnosis continued smokers)

Smoking status	N	Years (SD)	Outcome (%)	HR (95% CI)*	aHR (95% CI)†
First clinical disease worsening					
Non-smokers	1311	6.8 (4.5)	671 (50)	1.0 (reference)	1.0 (reference)
Quitters	72	7.4 (5.1)	39 (54)	1.01 (0.73 to 1.40)	1.01 (0.73 to 1.39)
Continued smokers	315	6.5 (4.7)	168 (53)	1.13 (0.96 to 1.34)	1.21 (1.03 to 1.44)
EDSS 3					
Non-smokers	1048	8.2 (4.7)	334 (32)	1.0 (reference)	1.0 (reference)
Quitters	63	9.0 (5.2)	21 (33)	0.96 (0.61 to 1.49)	0.95 (0.61 to 1.50)
Continued smokers	249	7.9 (4.9)	109 (44)	1.42 (1.15 to 1.77)	1.45 (1.16 to 1.81)
EDSS 4					
Non-smokers	1048	9.6 (4.4)	153 (15)	1.0 (reference)	1.0 (reference)
Quitters	63	10.5 (4.8)	8 (13)	0.80 (0.41 to 1.63)	0.90 (0.49 to 1.65)
Continued smokers	249	9.8 (4.5)	47 (19)	1.28 (0.92 to 1.77)	1.40 (1.01 to 1.96)
Physical worsening (increased MSIS-29 physical score by 7.5 or more)					
Non-smokers	1058	5.6 (4.3)	425 (40)	1.0 (reference)	1.0 (reference)
Quitters	56	5.8 (4.2)	28 (50)	1.23 (0.84 to 1.81)	1.23 (0.83 to 1.82)
Continued smokers	244	6.0 (6.7)	118 (48)	1.21 (0.99 to 1.49)	1.26 (1.02 to 1.56)
Psychological worsening (increased MSIS-29 psychological score by 7.5 or more)					
Non-smokers	1056	5.3 (4.4)	512 (48)	1.0 (reference)	1.0 (reference)
Quitters	56	5.8 (4.4)	30 (54)	1.03 (0.71 to 1.49)	1.05 (0.72 to 1.52)
Continued smokers	242	5.1 (6.6)	149 (62)	1.42 (1.18 to 1.71)	1.52 (1.26 to 1.83)
Cognitive disability worsening (decreased SDMT Score by 8 or more)					
Non-smokers	1079	5.9 (3.3)	287 (27)	1.0 (reference)	1.0 (reference)
Quitters	57	5.4 (3.4)	16 (28)	1.14 (0.69 to 1.89)	1.14 (0.68 to 1.91)
Continued smokers	252	6.1 (3.5)	80 (32)	1.16 (0.91 to 1.49)	1.40 (1.09 to 1.81)

*Crude.

†Adjusted for age at diagnosis, sex, disease phenotype, disease duration, baseline EDSS, disease-modifying therapy, passive smoking and snuff use.

EDSS, Expanded Disability Status Scale; MSIS-29, Multiple Sclerosis Impact Scale 29; SDMT, Symbol Digit Modalities Test.

### Supplementary analyses

All findings remained almost identical when we used January 2020 as the end of follow-up (data not shown). They also remained significant when we instead considered tobacco habits at the time of disease onset (data not shown), when we performed the analysis restricted to participants who had started treatment with disease-modifying drugs within 6 months after diagnosis (data not shown), and when we performed the analyses based on participants from EIMS and GEMS separately (data not shown).

## Discussion

Apart from active smoking, exposure to passive smoking appears to negatively influence disease activity and progression in MS. We replicated previous findings of smoking as a risk factor in MS, and therefore that smoking cessation after diagnosis is an important secondary preventive measure. Snuff use was not associated with higher disease activity or progression, suggesting that nicotine replacement therapy could be a safe way to increase the chance of quitting smoking among patients with MS.^23 24^

Accumulating evidence suggests a pathophysiological role of smoking in MS progression, and potential mechanisms have been discussed in detail elsewhere.^1 25^ Shortly, the association between smoking and faster MS disease progression may involve direct toxicity to neural tissue and modulation of immune responses with induction of a long-term inflammatory response, immune suppression, alteration of cytokine balance and epigenetic modifications.^1 25^ Another hypothesis, supported by experimental evidence,^26^ is that unspecific lung irritation is pivotal, since it is not nicotine itself that is detrimental, in view of the associated protection by snuff use. Although current dogma suggests that progressive disease is driven by a compartmentalised inflammation in the central nervous system, there is also evidence that systemic inflammation contributes to progression in form of interleukin 17 producing follicular T cells,^27^ which would be consistent with the speculation of a continuous lung irritation having a role in the progressive disease phase. These proposed mechanisms are also relevant regarding the impact of passive smoking on MS disease progression.

Smokers at baseline who stopped smoking post diagnosis did not significantly differ regarding disease outcomes compared with non-smokers at baseline, suggesting that the detrimental influence of smoking abates after smoking cessation. Although rather few patients stopped smoking post diagnosis, and the ability to assess benefits of smoking cessation was limited, our finding is in accordance with previous studies.^6–8^ A reduction of systemic inflammation and oxidative stress following smoking cessation may contribute to the favourable effect on disease outcomes.^28–30^

Although we found a significant influence of current smoking on the risk of cognitive disability worsening in MS as well as a trend showing increased risk of unfavourable outcome with increasing cumulative dose of smoking, we observed no significant association in our main analysis where individuals were followed up to 15 years. Further studies are thus needed to investigate the impact of smoking on cognitive functioning in MS.

An inverse dose–response correlation has been shown between cumulative dose of snuff use and subsequent MS risk^12^ and snuff use also appears to have beneficial effects on the progression of the disease. Tobacco contains numerous substances, and our study cannot formally rule out any of them as responsible for the current observation; however, nicotine is the most likely compound, in view of its active anti-inflammatory role. Nicotine exerts anti-inflammatory and immune-modulating effects through a mechanism dependent on the alpha7 nicotinic receptor^31^ and has been shown to suppress neuroinflammation and disease severity in experimental autoimmune encephalomyelitis.^32–35^

The lower HR of unfavourable outcomes among past snuff users than among current snuff users in our main analysis may be explained by the proportion of participants who replaced smoking with snuff use after the first clinical symptoms of the disease. Those who initiated snuff use close to diagnosis were predominantly past smokers at diagnosis (71%) whereas another 22% were current smokers. Pure nicotine substitutes were used by less than 0.4% of the cases in our study and meaningful comparisons were not possible.

Considering the negative impact of smoking on disease progression, smoking cessation is crucial. However, more than 80% of those who were smokers at MS diagnosis remained smokers 5 years post diagnosis. Qualitative studies have found low awareness among patients with MS regarding the adverse effects of smoking on the disease progression, and several MS-specific barriers to smoking cessation have been identified.^9–11^ Efforts should thus be made to better understand patient-specific barriers to stopping smoking and provide smoking cessation support for patients with MS. Nicotine replacement therapy could be part of such cessation support.

Experimental data in combination with our finding that users of moist snuff have a more favourable EDSS progression also suggest a possible strategy to affect MS progression. It should be noted that the use of nicotine as a therapeutic tool has potential disadvantages such as toxicity related side effects, lack of pharmacological specificity and unknown long-term effects on human health. However, selective agonists for the alpha7 nicotinic receptor could represent a pharmacological strategy to reduce neuroinflammation and neurodegeneration and may prove beneficial in patients with MS.^36^

A strength of our study is the population-based design, the high response rate, and the detailed information regarding exposures which makes it possible to consider several potential confounding factors. The response rate was lower in the EIMS follow-up study; however, there were no significant differences in baseline EDSS or at 5-year post diagnosis among those who participated in the follow-up study and those who did not.

Information regarding smoking and snuff use habits in EIMS was collected at baseline and should be subjected to limited recall bias, whereas information regarding lifestyle habits was collected retrospectively for patients included in GEMS. Some prevalent cases may also have suffered from cognitive impairment. However, although the risk of potential recall bias regarding self-reported lifestyle habits is higher in GEMS, we observed similar results when EIMS and GEMS participants were analysed separately.

Self-reported tobacco consumption habits may to some extent be under-reported. The objective method to distinguish subjects by tobacco consumption habits is the measurement of cotinine, the main metabolite of nicotine. However, it is not possible to distinguish smokers from snuff users by cotinine levels and self-reported tobacco consumption habits are thus preferable in this context.^37^ Of those who had been classified as exposed to passive smoking, 87% had been exposed at home. We did not have information regarding the frequency of passive smoking, except from time periods when it occurred daily. We also did not have information regarding the number of people who smoked or the size of the home/workplace.

In conclusion, both current smoking and current exposure to passive smoking negatively impacts disease progression in patients with MS. Our finding that snuff use is not associated with worse disease progression indicates that nicotine replacement therapy could be an attractive way to increase the chance of quitting smoking post diagnosis.

## Data Availability

Data are available upon reasonable request. Anonymised data underlying this article will be shared on reasonable request from any qualified investigator that wants to analyse questions that are related to the published article.
