# Supplementary material for: Influence of oral tobacco versus smoking on multiple sclerosis disease activity and progression
Source: J Neurol Neurosurg Psychiatry. 2023 Mar 31;94(8):589–96. doi: 10.1136/jnnp-2022-330848 (PMC10359558; doi:10.1136/jnnp-2022-330848)
Supplement: Supplementary data [file jnnp-2022-330848supp001.pdf]

Supplementary Methods

The model below was used to predict the variation of MS progression scores by a quadratic specification of the time of follow-up:

MS progression scores$_{ij}$ = $\beta_0 + \beta_1 t_{ij} + \beta_2 t_{ij}^2 + \beta_3$smoking $+ \beta_4$smoking $t_{ij} + \beta_5$smokingt_{ij}^2 + $\beta_6$covariates + $b_{1i} + b_{2i}t_{ij} + b_{3i}t_{ij}^2 + \varepsilon_{ij}$,

i= 1, 2, 3…n, j=1, 2, 3…15

where MS progression scores$_{ij}$ represents the estimated mean of annual MS progression scores for individual i at time = j (in years, ranging from 1 to 15 years); n is the sample size; β_0 represents the intercept (thus MS progression scores at baseline, when t_{ij} =0); β_1 and β_2 are the estimated linear and quadratic MS progression scores change associated with the change from time point j to end of follow-up; β_3 is the intercept of MS progression scores by smoking status; β_4 and β_5 are the estimated linear and quadratic terms for the rate of MS progression scores change (slope) by smoking status (never smoking as reference); b_{0i} represents the random effect for individual i, b_{1i} and b_{2i} represent the random effect for the slope respectively.

Supplementary table 1. Baseline characteristics of overall sample and by smoking status at diagnosis.

	Total	Never smoker	Current smoking	Past smoking	P value
N	9,089	3,675	2,897	2,517	
Age at diagnosis (SD)	37.6 (11.1)	36.5 (11.0)	35.9 (10.6)	41.1 (10.9)	<0.0001
Female, n (%)	6541 (72)	2669 (73)	2073 (72)	1851 (71)	0.51
Nordic origin, n (%)	7796 (86)	3164 (87)	2465 (86)	2167 (87)	0.31
Treatment, n (%)	7488 (82)	3116 (85)	2303 (80)	2069 (82)	<0.0001
MS phenotype<br>Relapsing onset, n (%)<br>Progressive onset, n (%)<br>Unknown, n (%)	 8267 (916)<br>725 (8.0)<br>97 (1.1)	 3373 (92)<br>254 (6.9)<br>48 (1.3)	 2643 (91)<br>224 (7.7)<br>30 (1.0)	 2251 (89)<br>247 (9.8)<br>19 (0.8)	<0.0001
Disease duration at diagnosis, years (SD)	6.1 (6.8)	5.7 (6.4)	5.8 (6.3)	7.0 (7.6)	<0.0001
Baseline EDSS (SD)	2.8 (2.2)	2.6 (2.2)	3.2 (2.3)	2.7 (2.2)	<0.0001
Baseline MSIS-PHYS (SD)	25 (23)	22 (22)	30 (25)	25 (23)	<0.0001
Baseline MSIS-PSYCH (SD)	31 (24)	28 (23)	35 (25)	32 (23)	<0.0001
Baseline SDMT (SD)	50 (13)	51 (13)	47 (13)	49 (13)	<0.0001
Pack years of smoking (SD)	4.6 (8.1)	0	9.3 (9.8)	6.1 (8.2)	<0.0001
Passive smoking, n (%)	5576 (61)	1877 (51)	2011 (69)	1688 (67)	<0.0001
Snuff use, n (%)	1568 (17)	315 (8.6)	635 (22)	618 (25)	<0.0001
Alcohol (gram/week, SD)	31 (45)	26 (38)	36 (51)	35 (45)	<0.0001
Body mass index, kg/m^2 (SD)	25.0 (4.8)	24.9 (4.7)	25.0 (4.7)	25.3 (5.1)	0.001

Supplementary table 2. Baseline characteristics of overall sample and by passive smoking status at diagnosis.

	Total	Never exposed	Current exposure	Past exposure	P value
N	9,089	3,513	1,177	4,399	
Age at diagnosis (SD)	37.6 (11.1)	35.4 (11.0)	34.9 (11.0)	40.1 (10.6)	<0.0001
Female, n (%)	6541 (72)	2476 (70)	799 (68)	3266 (74)	<0.0001
Nordic origin, n (%)	7796 (86)	3034 (87)	1001 (86)	3761 (86)	0.31
Treatment, n (%)	7488 (82)	3017 (86)	787 (67)	3684 (84)	<0.0001
MS phenotype<br>Relapsing onset, n (%)<br>Progressive onset, n (%)<br>Unknown, n (%)	 8267 (916)<br>725 (8.0)<br>208 (2.2)	 3242 (92)<br>234 (6.7)<br>37 (1.1)	 1062 (90)<br>104 (8.8)<br>11 (0.9)	 3963 (90)<br>387 (8.8)<br>49 (1.1)	0.003
Disease duration at diagnosis, years (SD)	6.1 (6.8)	5.6 (6.5)	6.1 (6.4)	6.4 (7.0)	<0.0001
Baseline EDSS (SD)	2.8 (2.2)	2.5 (2.2)	4.0 (2.5)	2.7 (2.1)	<0.0001
Baseline MSIS-PHYS (SD)	225 (23)	22 (22)	33 (25)	27 (23)	<0.0001
Baseline SDMT (SD)	50 (13)	51 (13)	44 (14)	49 (13)	<0.0001
Ever smoking, n (%)	5414 (60)	1715 (49)	902 (77)	2797 (64)	<0.0001
Pack years of smoking (SD)	4.6 (8.1)	2.7 (6.1)	6.8 (9.3)	5.5 (8.7)	<0.0001
Snuff use, n (%)	1568 (17)	589 (17)	216 (18)	763 (17)	0.44
Alcohol (gram/week, SD)	31 (45)	31 (44)	29 (47)	33 (45)	<0.0001
Body mass index, kg/m^2 (SD)	25.0 (4.8)	24.5 (4.4)	25.0 (4.9)	25.4 (5.0)	<0.0001

Supplementary table 3. Baseline characteristics of overall sample and by snuff use status at diagnosis.

	Total	Never exposed	Current exposure	Past exposure	P value
N	9,089	7,540	1,097	452	
Age at diagnosis (SD)	37.6 (11.1)	37.9 (11.3)	35.0 (9.9)	38.9 (9.8)	<0.0001
Female, n (%)	6541 (72)	5966 (79)	402 (37)	162 (36)	<0.0001
Nordic origin, n (%)	7796 (86)	6420 (86)	963 (89)	395 (95)	<0.0001
Treatment, n (%)	7488 (82)	6102 (81)	971 (89)	399 (88)	<0.0001
MS phenotype<br>Relapsing onset, n (%)<br>Progressive onset, n (%)<br>Unknown, n (%)	 8267 (91)<br>725 (8.0)<br>208 (2.2)	 6839 (91)<br>592 (7.9)<br>90 (1.2)	 1004 (92)<br>88 (8.0)<br>5 (0.5)	 409 (90)<br>41 (9.1)<br>2 (0.4)	0.29
Disease duration at diagnosis, years (SD)	6.1 (6.8)	6.2 (6.9)	5.4 (5.5)	5.6 (6.6)	<0.0001
Baseline EDSS (SD)	2.8 (2.2)	2.9 (2.3)	2.7 (2.2)	2.6 (2.1)	0.05
Baseline MSIS-PHYS (SD)	225 (23)	25 (23)	26 (23)	23 (22)	0.06
Baseline SDMT (SD)	50 (13)	50 (13)	48 (13)	51 (12)	<0.0001
Snuff use duration, years (SD)	2.0 (6.3)	0	9.4 (9.7)	17.8 (10.4)	<0.0001
Ever smoking, n (%)	5414 (60)	4161 (55)	882 (80)	359 (79)	<0.0001
Pack years of smoking (SD)	4.6 (8.1)	4.3 (7.7)	6.4 (8.1)	6.2 (9.9)	<0.0001
Passive smoking, n (%)	5567 (61)	4597 (61)	678 (62)	292 (65)	0.32
Alcohol (gram/week, SD)	35 (73)	32 (72)	51 (72)	54 (79)	<0.0001
Body mass index, kg/m^2 (SD)	25.0 (4.8)	25.0 (4.9)	25.2 (4.5)	25.4 (4.5)	0.0009

Supplementary table 4. β coefficients and 95% CIs of the associations between active/passive smoking and MS progression over 15 years using mixed-effect models.

	EDSS	MSIS-PHYS	SDMT
	**β (95%CI)**	**β (95%CI)**	**β (95%CI)**
**Smoking status**			
Never smoker	Ref	Ref	Ref
Past smoker	-0.06 (-0.17, 0.05)	1.21 (-0.02, 2.44)	-0.46 (-1.35, 0.43)
Current smoker	0.48 (0.38, 0.58)	6.61 (5.43, 7.80)	-3.64 (-4.50, -2.78)
Never smoker x time	Ref	Ref	Ref
Past smoker x time	0.02 (0.01, 0.03)	0.01 (-0.25, 0.27)	-0.27 (-0.43, -0.10)
Current smoker x time	0.03 (0.02, 0.04)	0.03 (-0.22, 0.28)	-0.004 (-0.16, 0.15)
Never smoker x $time^2$	Ref	Ref	Ref
Past smoker x $time^2$	-0.001 (-0.002, 0.000)	-0.006(-0.039, 0.026)	0.005 (-0.020, 0.029)
Current smoker x $time^2$	-0.001 (-0.002, 0.000)	-0.019(-0.049, 0.012)	-0.002 (-0.025, 0.022)
**Passive smoking**			
Never exposed	Ref	Ref	Ref
Past exposure	0.05 (-0.04, 0.15)	2.00 (0.93, 3.06)	-0.37 (-1.13, 0.40)
Current exposure	1.23 (1.09, 1.37)	7.67 (5.70, 9.64)	-5.83 (-7.27, -4.40)
Never exposed x time	Ref	Ref	Ref
Past exposure x time	0.01 (0.001, 0.02)	0.12 (-0.10, 0.02)	-0.49 (-0.63, -0.36)
Current exposure x time	0.04 (0.03, 0.06)	0.23 (-0.19, 0.64)	-0.07 (-0.33, 0.19)
Never exposed x $time^2$	Ref	Ref	Ref
Past exposure x $time^2$	-0.000 (-0.001, 0.001)	-0.012 (-0.039, 0.015)	0.045 (0.025, 0.066)
Current exposure x $time^2$	-0.001(-0.003, 0.000)	-0.018 (-0.068, 0.032)	0.022 (-0.016, 0.060)

All analyses were adjusted for age at diagnosis, sex, disease phenotype, disease duration, baseline EDSS, and disease-modifying therapy (ever/never).

Supplementary table 5. Predicted mean outcome values at baseline, 3, 5, 10, and 15 years post-baseline, by smoking status and exposure to passive smoking status, respectively.

Predicted mean EDSS scores at baseline, 3, 5, 10 and 15 years of follow-up					
Smoking status	Baseline	Year 3	Year 5	Year 10	Year 15
Never smoker	2.43 (2.37-2.50)	2.72 (2.65-2.78)	2.89 (2.82-2.96)	3.26 (3.18-3.35)	3.56 (3.42-3.69)
Past smoker	2.55 (2.47-2.63)	2.90 (2.82-2.98)	3.11 (3.02-3.19)	3.55 (3.45-3.65)	3.87 (3.71-4.03)
Current smoker	2.96 (2.88-3.03)	3.31 (3.23-3.39)	3.52 (3.44-3.60)	3.95 (3.86-4.05)	4.25 (4.11-4.40)
Predicted mean MSIS-28 physical score at baseline, 3, 5, 10, and 15 years of follow-up					
Smoking status	Baseline	Year 3	Year 5	Year 10	Year 15
Never smoker	20.0 (19.2-20.7)	19.7 (19.0-20.5)	20.1 (19.4-20.9)	23.0 (21.8-24.2)	28.6 (25.8-31.4)
Past smoker	22.7 (21.8-23.7)	22.6 (21.6-23.5)	22.9 (21.9-23.9)	25.2 (23.6-26.9)	29.8 (26.0-33.7)
Current smoker	28.0 (27.1-28.9)	27.8 (27.0-28.9)	27.9 (27.0-28.9)	29.5 (28.0-31.0)	32.8 (29.3-36.2)
Predicted mean SDMT score at baseline, 3, 5, 10 and 15 years of follow-up					
Smoking status	Baseline	Year 3	Year 5	Year 10	Year 15
Never smoker	51.8 (51.3-52.4)	56.9 (56.4-57.4)	57.4 (56.8-57.9)	48.1 (47.0-49.1)	24.0 (21.6-26.4)
Past smoker	49.9 (49.2-50.6)	54.2 (53.5-54.9)	54.2 (53.5-54.9)	43.9 (42.5-45.3)	19.1 (15.9-22.4)
Current smoker	47.6 (47.0-48.3)	52.7 (52.0-53.4)	53.1 (52.4-53.8)	43.7 (42.4-45.0)	19.4 (16.5-22.4)
Predicted mean EDSS scores at baseline, 3, 5, 10 and 15 years of follow-up					
Passive smoking	Baseline	Year 3	Year 5	Year 10	Year 15
Never exposed	2.36 (2.30-2.43)	2.66 (2.59-2.73)	2.84 (2.77-2.91)	3.22 (3.14-3.31)	3.51 (3.38-3.64)
Past exposure	2.56 (2.50-2.62)	2.88 (2.82-2.94)	3.08 (3.01-3.14)	3.49 (3.41-3.56)	3.78 (3.66-3.90)
Current exposure	3.83 (3.71-3.95)	4.24 (4.12-4.36)	4.49 (4.36-4.61)	4.99 (4.85-5.14)	5.35 (5.12-5.58)
Predicted mean MSIS-28 physical score at baseline, 3, 5, 10, and 15 years of follow-up					
Passive smoking	Baseline	Year 3	Year 5	Year 10	Year 15
Never exposed	20.1 (19.3-20.8)	19.7 (19.0-20.5)	20.0 (19.2-20.8)	22.5 (21.3-23.7)	27.6 (24.8-30.3)
Past exposure	24.6 (23.9-25.3)	24.4 (23.7-25.1)	24.8 (24.0-25.5)	27.0 (25.8-28.2)	31.3 (28.4-34.1)
Current exposure	30.8 (29.0-32.6)	30.8 (29.0-32.6)	31.2 (29.3-33.1)	33.6 (30.5-36.6)	37.9 (31.1-44.7)
Predicted mean SDMT score at baseline, 3, 5, 10 and 15 years of follow-up					
Passive smoking	Baseline	Year 3	Year 5	Year 10	Year 15
Never exposed	51.5 (51.0-52.1)	56.9 (56.4-57.5)	57.3 (56.8-57.9)	47.2 (46.2-48.3)	21.3 (18.9-23.6)
Past exposure	49.7 (49.1-50.2)	54.0 (53.5-54.5)	54.1 (53.6-54.7)	44.9 (43.9-46.0)	22.1 (19.7-24.5)
Current exposure	44.6 (43.3-46.0)	50.0 (48.7-51.3)	50.6 (49.2-52.0)	41.8 (39.2-44.4)	18.1 (12.3-24.0)

All analyses were adjusted for age at diagnosis, sex, disease phenotype, disease duration, baseline EDSS, and disease-modifying therapy.

Supplementary 6. HR with 95% CI of having unfavorable outcomes post-diagnosis, by smoking habits at diagnosis.

First clinical disease worsening (CDW)					
	Total	Women		Men	
Smoking status	aHR (95% CI)[1]	HR (95% CI)[2]	aHR (95% CI)[1]	HR (95% CI)[2]	aHR (95% CI)[1]
Never exposed	1.0 (reference)	1.0 (reference)	1.0 (reference)	1.0 (reference)	1.0 (reference)
Past smoking	1.01 (0.94-1.08)	1.02 (0.94-1.10)	1.01 (0.90-1.05)	1.12 (0.99-1.26)	1.08 (0.94-1.23)
Current smoking	1.13 (1.06-1.21)	1.13 (1.05-1.22)	1.13 (1.05-1.22)	1.15 (1.01-1.31)	1.13 (1.00-1.27)
EDSS 3					
Smoking status	aHR (95% CI)[1]	HR (95% CI)[2]	aHR (95% CI)[1]	HR (95% CI)[2]	aHR (95% CI)[1]
Never smoking	1.0 (reference)	1.0 (reference)	1.0 (reference)	1.0 (reference)	1.0 (reference)
Past smoking	1.00 (0.90-1.12)	1.08 (0.96-1.22)	0.99 (0.86-1.10)	1.20 (0.98-1.47)	1.07 (0.86-1.33)
Current smoking	1.21 (1.09-1.34)	1.22 (1.09-0.37)	1.19 (1.05-1.34)	1.26 (1.02-1.54)	1.28 (1.03-1.59)
EDSS 4					
Smoking status	aHR (95% CI)[1]	HR (95% CI)[2]	aHR (95% CI)[1]	HR (95% CI)[2]	aHR (95% CI)[1]
Never smoking	1.0 (reference)	1.0 (reference)	1.0 (reference)	1.0 (reference)	1.0 (reference)
Past smoking	1.06 (0.92-1.23)	1.02 (0.86-1.21)	0.94 (0.78-1.08)	1.18 (0.91-1.55)	1.08 (0.83-1.44)
Current smoking	1.28 (1.11-1.45)	1.25 (1.07-1.46)	1.28 (1.09-1.50)	1.34 (1.03-1.74)	1.42 (1.08-1.86)
Physical worsening (increased MSIS-29 physical score by 7.5 or more)					
Smoking status	aHR (95% CI)[1]	HR (95% CI)[2]	aHR (95% CI)[1]	HR (95% CI)[2]	aHR (95% CI)[1]
Never smoking	1.0 (reference)	1.0 (reference)	1.0 (reference)	1.0 (reference)	1.0 (reference)
Past smoking	1.06 (0.95-1.19)	1.07 (0.95-1.22)	1.03 (0.91-1.18)	1.17 (0.98-1.46)	1.13 (0.91-1.40)
Current smoking	1.23 (1.11-1.37)	1.18 (1.05-1.33)	1.22 (1.08-1.38)	1.19 (0.96-1.44)	1.25 (1.01-1.54)
Psychological worsening (increased MSIS-29 psychological score by 7.5 or more)					
Smoking status	aHR (95% CI)[1]	HR (95% CI)[2]	aHR (95% CI)[1]	HR (95% CI)[2]	aHR (95% CI)[1]
Never smoking	1.0 (reference)	1.0 (reference)	1.0 (reference)	1.0 (reference)	1.0 (reference)
Past smoking	1.18 (1.07-1.31)	1.05 (0.94-1.18)	1.11 (0.98-1.24)	1.39 (1.12-1.63)	1.44 (1.18-1.76)
Current smoking	1.27 (1.15-1.40)	1.13 (1.01-1.26)	1.21 (1.08-1.36)	1.35 (1.15-1.68)	1.44 (1.19-1.76)
Cognitive disability worsening (decreased SDMT score by 8 or more)					
Smoking status	aHR (95% CI)[1]	HR (95% CI)[2]	aHR (95% CI)[1]	HR (95% CI)[2]	aHR (95% CI)[1]
Never smoking	1.0 (reference)	1.0 (reference)	1.0 (reference)	1.0 (reference)	1.0 (reference)
Past smoking	1.08 (0.93-1.25)	0.99 (0.84-1.16)	1.14 (0.96-1.35)	0.96 (0.72-1.23)	0.92 (0.68-1.23)
Current smoking	1.17 (1.02-1.36)	1.22 (1.04-1.43)	1.16 (0.98-1.38)	1.05 (0.81-1.38)	1.21 (0.91-1.60)

[1]adjusted for age at diagnosis, disease phenotype, disease duration, baseline EDSS, disease-modifying therapy, passive smoking, and snuff use; [2]crude.

Supplementary table 7. HR with 95% CI of having unfavorable outcomes post-diagnosis, by passive smoking status at diagnosis. Restricted to **never-smokers** at diagnosis.

First clinical disease worsening (CDW)					
Passive smoking	N	Years (SD)	Outcome (%)	HR (95% CI)[1]	HR (95% CI)[2]
Never exposed	1798	6.6 (4.9)	991 (55)	1.0 (reference)	1.0 (reference)
Past exposure	1602	6.3 (4.9)	874 (55)	1.03 (0.93-1.12)	0.99 (0.90-1.08)
Current exposure	275	6.4 (5.1)	166 (60)	1.19 (1.01-1.40)	1.17 (1.00-1.38)
EDSS 3					
Passive smoking	N	Years (SD)	Outcome (%)	HR (95% CI)	HR (95% CI)
Never exposed	1173	8.2 (5.3)	451 (38)	1.0 (reference)	1.0 (reference)
Past exposure	1003	7.5 (5.2)	363 (36)	1.00 (0.87-1.14)	1.00 (0.81-1.07)
Current exposure	105	7.1 (5.0)	53 (50)	1.48 (1.12-1.97)	1.38 (1.04-1.84)
EDSS 4					
Passive smoking	N	Years (SD)	Outcome (%)	HR (95% CI)	HR (95% CI)
Never exposed	1173	9.7 (5.3)	248 (21)	1.0 (reference)	1.0 (reference)
Past exposure	1003	9.4 (5.4)	184 (18)	0.95 (0.78-1.14)	0.94 (0.73-1.06)
Current exposure	105	9.5 (4.9)	34 (32)	1.65 (1.15-2.36)	1.52 (1.06-2.18)
Physical worsening (increased MSIS-29 score by 7.5 or more)					
Passive smoking	N	Years (SD)	Outcome (%)	HR (95% CI)	HR (95% CI)
Never exposed	1113	5.3 (4.4)	469 (42)	1.0 (reference)	1.0 (reference)
Past exposure	912	4.8 (4.5)	376 (41)	1.06 (0.93-1.22)	1.02 (0.89-1.17)
Current exposure	89	4.4 (4.5)	46 (52)	1.54 (1.13-2.08)	1.63 (1.20-2.21)
Psychological worsening (increased MSIS-29 psych score by 7.5 or more)					
Passive smoking	N	Years (SD)	Outcome (%)	HR (95% CI)	HR (95% CI)
Never exposed	1108	4.9 (4.4)	550 (50)	1.0 (reference)	1.0 (reference)
Past exposure	912	7.8 (4.4)	440 (48)	1.05 (0.92-1.19)	1.12 (0.99-1.28)
Current exposure	89	4.3 (4.3)	44 (49)	1.19 (0.87-1.61)	1.31 (0.96-1.78)
Cognitive disability worsening (decreased SDMT score by 8 or more)					
Passive smoking	N	Years (SD)	Outcome (%)	HR (95% CI)	HR (95% CI)
Never exposed	1177	5.6 (3.5)	264 (22)	1.0 (reference)	1.0 (reference)
Past exposure	944	5.2 (3.5)	212 (22)	1.09 (0.91-1.31)	0.99 (0.82-1.19)
Current exposure	96	5.9 (3.8)	28 (29)	1.39 (0.94-2.05)	1.69 (1.13-2.47)

crude; adjusted for age at diagnosis, sex, disease phenotype, disease duration, baseline EDSS, disease-modifying therapy, and snuff use.

Supplementary table 8. HR with 95% CI of having unfavorable outcomes post-diagnosis, by passive smoking status at diagnosis. Restricted to those with **disease modifying treatment**.

First clinical disease worsening (CDW)					
Passive smoking	N	Years (SD)	Outcome (%)	HR (95% CI)[1]	HR (95% CI)[2]
Never exposed	3017	6.7 (5.0)	1718 (57)	1.0 (reference)	1.0 (reference)
Past exposure	3684	6.3 (4.9)	2149 (58)	1.10 (1.03-1.17)	1.03 (0.97-1.10)
Current exposure	787	6.4 (5.1)	528 (67)	1.29 (1.17-1.43)	1.25 (1.13-1.39)
EDSS 3					
Passive smoking	N	Years (SD)	Outcome (%)	HR (95% CI)	HR (95% CI)
Never exposed	2073	8.3 (5.3)	792 (38)	1.0 (reference)	1.0 (reference)
Past exposure	2350	7.6 (5.3)	977 (42)	1.18 (1.07-1.30)	1.04 (0.94-1.14)
Current exposure	342	7.2 (5.4)	195 (57)	1.70 (1.45-2.0)	1.56 (1.33-1.82)
EDSS 4					
Passive smoking	N	Years (SD)	Outcome (%)	HR (95% CI)	HR (95% CI)
Never exposed	2073	9.8 (5.4)	435 (21)	1.0 (reference)	1.0 (reference)
Past exposure	2350	9.6 (5.5)	498 (21)	1.07 (0.94-1.22)	0.97 (0.88-1.05)
Current exposure	342	9.3 (5.6)	133 (39)	2.01 (1.66-2.44)	1.63 (1.41-2.11)
Physical worsening (increased MSIS-29 physical score by 7.5 or more)					
Passive smoking	N	Years (SD)	Outcome (%)	HR (95% CI)	HR (95% CI)
Never exposed	2029	5.4 (4.5)	875 (43)	1.0 (reference)	1.0 (reference)
Past exposure	2306	4.9 (4.7)	1012 (44)	1.12 (1.02-1.23)	1.05 (0.96-1.16)
Current exposure	340	4.5 (4.3)	172 (51)	1.42 (1.20-1.67)	1.45 (1.23-1.72)
Psychological worsening (increased MSIS-29 psychological score by 7.5 or more)					
Passive smoking	N	Years (SD)	Outcome (%)	HR (95% CI)	HR (95% CI)
Never exposed	2025	5.0 (4.5)	1041 (51)	1.0 (reference)	1.0 (reference)
Past exposure	2296	4.5 (4.7)	1164 (51)	1.08 (0.99-1.17)	1.13 (1.04-1.24)
Current exposure	336	4.2 (4.3)	189 (56)	1.29 (1.11-1.51)	1.41 (1.21-1.65)
Cognitive disability worsening (decreased SDMT score by 8 or more)					
Passive smoking	N	Years (SD)	Outcome (%)	HR (95% CI)	HR (95% CI)
Never exposed	2134	5.8 (3.5)	500 (23)	1.0 (reference)	1.0 (reference)
Past exposure	2403	5.3 (3.5)	567 (24)	1.12 (0.99-1.26)	1.03 (0.91-1.17)
Current exposure	356	5.7 (3.7)	78 (22)	0.99 (0.78-1.26)	1.17 (0.92-1.49)

[1]crude; [2]adjusted for age at diagnosis, sex, disease phenotype, disease duration, baseline EDSS, disease-modifying therapy, smoking, and snuff use.

Supplementary table 8. HR with 95% CI of having unfavorable outcomes post-diagnosis, by snuff use habits at diagnosis. Restricted to those with **disease modifying treatment**.

First clinical disease worsening (CDW)					
Snuff use status	N	Years (SD)	Outcome (%)	HR (95% CI)[1]	HR (95% CI)[2]
Never user	6102	6.4 (5.0)	3603 (59)	1.0 (reference)	1.0 (reference)
Past user	415	6.6 (5.0)	215 (51)	0.85 (0.74-0.98)	0.82 (0.71-0.94)
Current user	971	6.4 (5.0)	577 (59)	0.99 (0.91-1.09)	0.98 (0.90-1.08)
EDSS 3					
Snuff use status	N	Years (SD)	Outcome (%)	HR (95% CI)	HR (95% CI)
Never user	3906	7.9 (5.3)	1634 (42)	1.0 (reference)	1.0 (reference)
Past user	255	7.6 (5.2)	80 (32)	0.80 (0.64-1.00)	0.79 (0.63-0.99)
Current user	604	8.0 (5.6)	246 (41)	0.95 (0.83-1.08)	0.93 (0.80-1.07)
EDSS 4					
Snuff use status	N	Years (SD)	Outcome (%)	HR (95% CI)	HR (95% CI)
Never user	3906	9.8 (5.4)	891 (23)	1.0 (reference)	1.0 (reference)
Past user	255	9.0 (5.1)	48 (19)	0.88 (0.70-1.10)	0.80 (0.69-0.98)
Current user	604	9.9 (5.7)	127 (21)	0.99 (0.86-1.13)	0.89 (0.66-1.20)
Physical worsening (increased MSIS-29 physical score by 7.5 or more)					
Snuff use status	N	Years (SD)	Outcome (%)	HR (95% CI)	HR (95% CI)
Never user	3749	5.1 (4.6)	1672 (45)	1.0 (reference)	1.0 (reference)
Past user	276	5.4 (5.2)	123 (45)	0.95 (0.79-1.14)	0.91 (0.75-1.09)
Current user	646	5.0 (4.0)	262 (41)	0.91 (0.80-1.04)	0.91 (0.79-1.05)
Psychological worsening (increased MSIS-29 psychological score by 7.5 or more)					
Snuff use status	N	Years (SD)	Outcome (%)	HR (95% CI)	HR (95% CI)
Never user	3736	4.7 (4.6)	1929 (52)	1.0 (reference)	1.0 (reference)
Past user	276	4.8 (5.0)	144 (52)	0.99 (0.84-1.18)	0.98 (0.83-1.17)
Current user	641	4.5 (3.9)	318 (50)	0.97 (0.87-1.10)	0.97 (0.86-1.10)
Cognitive disability worsening (decreased SDMT score by 8 or more)					
Snuff use status	N	Years (SD)	Outcome (%)	HR (95% CI)	HR (95% CI)
Never user	3928	5.5 (3.6)	924 (24)	1.0 (reference)	1.0 (reference)
Past user	291	5.6 (3.4)	67 (23)	0.92 (0.72-1.18)	0.82 (0.64-1.06)
Current user	666	5.5 (3.6)	152 (23)	0.96 (0.81-1.14)	0.99 (0.83-1.19)

[1]crude; [2]adjusted for age at diagnosis, sex, disease phenotype, disease duration, baseline EDSS, disease-modifying therapy, smoking and passive smoking.
